# Supplementary material for: Force-clamp spectroscopy identifies a catch bond mechanism in a Gram-positive pathogen
Source: Nat Commun. 2020 Oct 27;11:5431. doi: 10.1038/s41467-020-19216-8 (PMC7591895; doi:10.1038/s41467-020-19216-8)
Supplement: Supplementary file 1 — Supplementary Informatioon [file 41467_2020_19216_MOESM1_ESM.pdf]

Supplementary Information for

Force-clamp spectroscopy identifies a catch bond mechanism in a Gram-positive pathogen

Mathelié-Guinlet et al.

This PDF file includes supplementary figures 1 to 8 and supplementary tables 1 to 3.

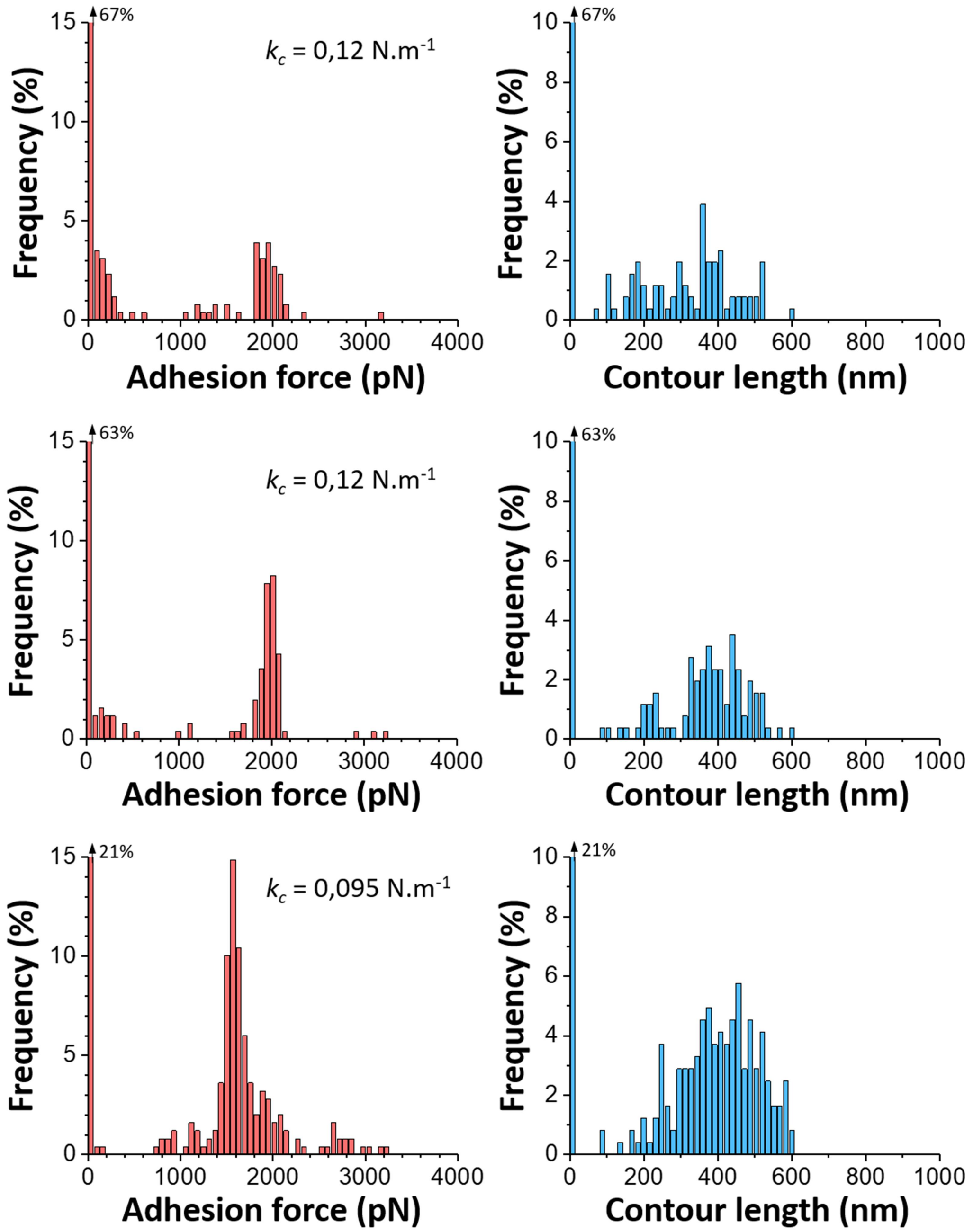

Supplementary Figure 1. Single-cell force spectroscopy of the SpsD-Fg interaction. Maximum adhesion force and contour length histograms, extracted from an extensible worm like chain model, and obtained between three other independent *S. pseudintermedius* ED99 SpsD cells and fibrinogen-coated surfaces. Experiments were performed at room temperature, in PBS buffer and at a retraction velocity of 1,000 nm.s^{-1}. The spring constant of the cantilever, k_c, used to probe each cell is specified in insets.

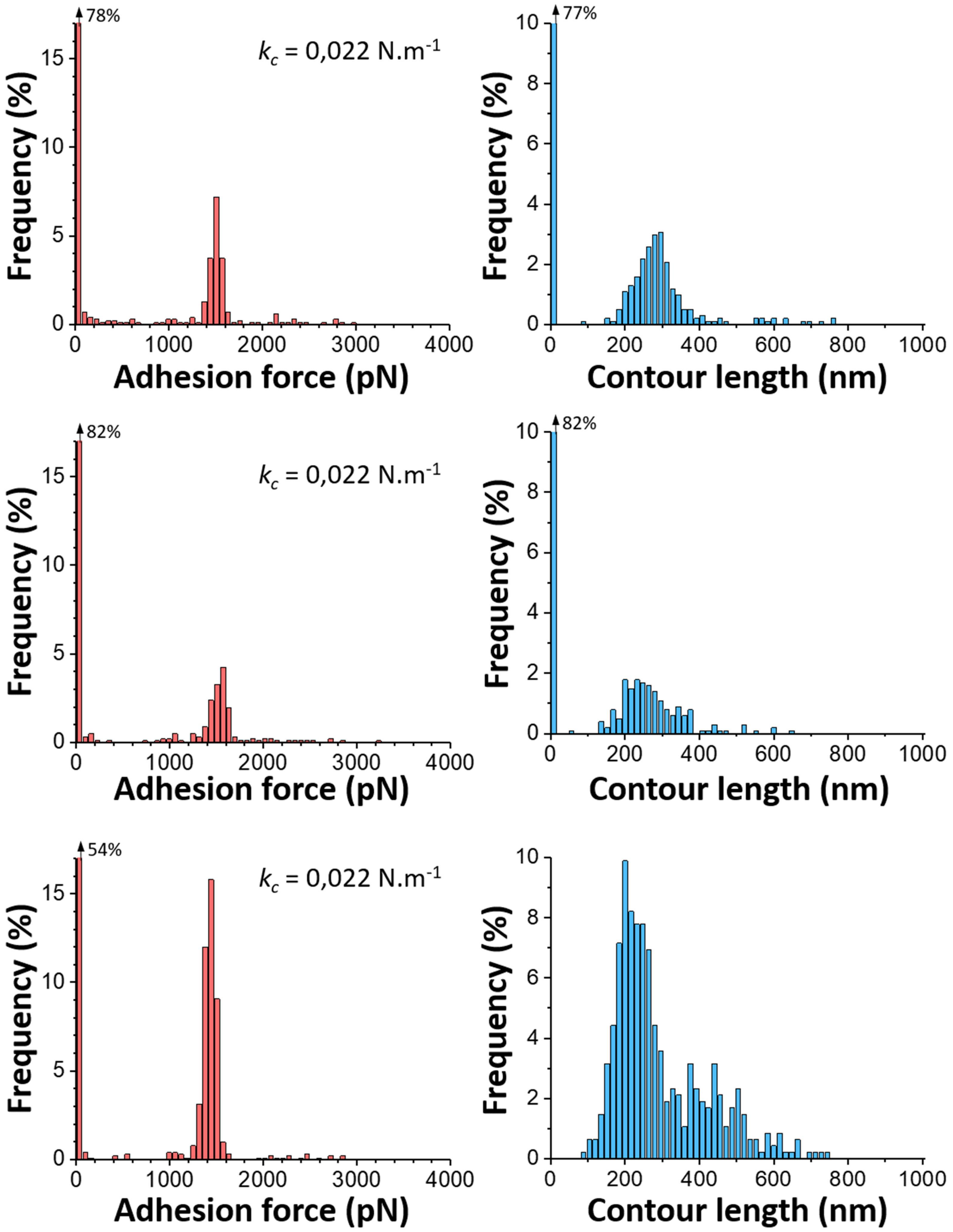

Supplementary Figure 2. Single-molecule force spectroscopy of the SpsD-Fg interaction. Maximum adhesion force and contour length histograms, extracted from an extensible worm like chain model, and obtained between three other independent *S. pseudintermedius* ED99 SpsD cells and AFM tips functionalized with fibrinogen. Experiments were performed at room temperature, in PBS buffer and at a retraction velocity of 1,000 nm.s^{-1}. The spring constant of the cantilever, k_c, used to probe each cell is specified in insets.

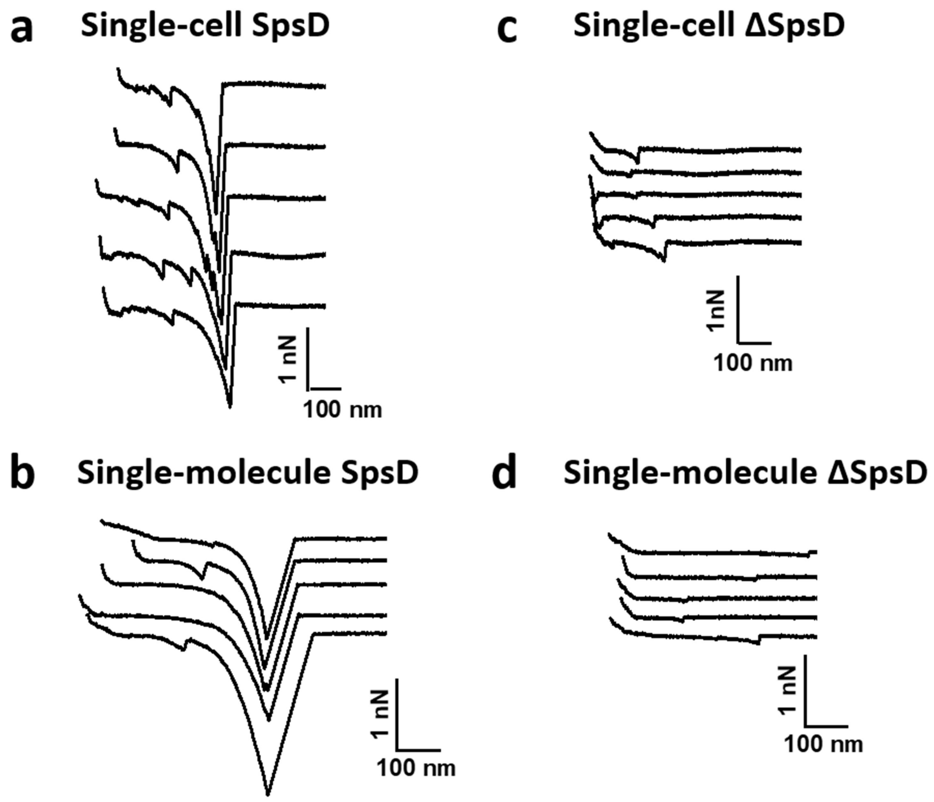

Supplementary Figure 3. Representative retraction profiles recorded in (a-b) single-cell and (c-d) single-molecule experiments between fibrinogen and *S. pseudintermedius* ED99 cells either (a,c) exhibiting only SpsD adhesins or (b,d) lacking such adhesins.

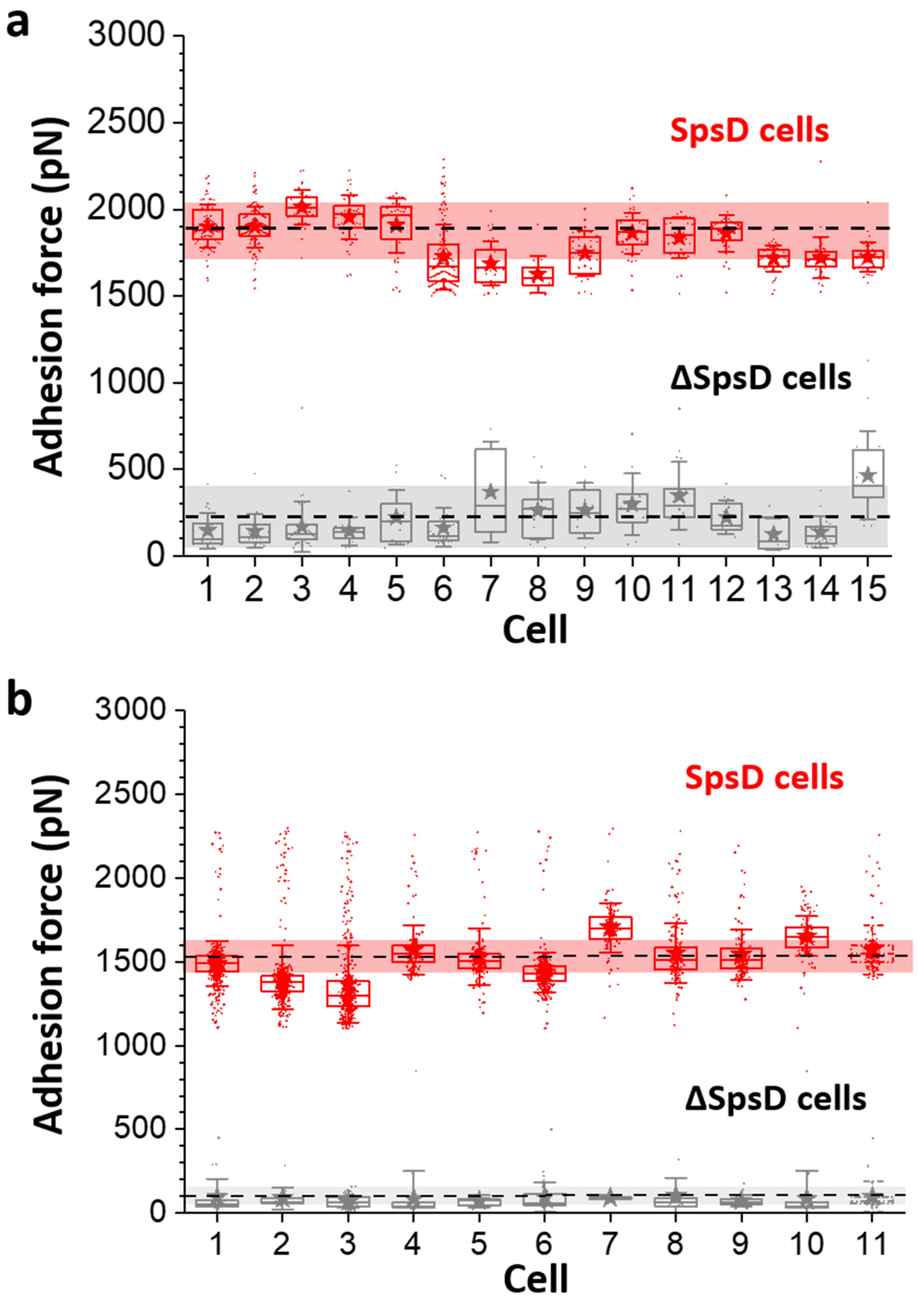

Supplementary Figure 4. Individual box plot per single cell (overlapped with data) of the adhesion forces observed in (a) single-cell and (b) single-molecule experiments on both SpsD and ΔSpsD cells. Stars are the mean values, lines the medians, boxes the 25-75 % quartiles and whiskers the standard deviation. The number of adhesive events for each cell depend on the adhesion probability. The dashed black lines (for both SpsD and ΔSpsD cells) stand for the mean adhesion force obtained from all the cells (*n* = 15 and *n* = 11 cells from 5 independent cultures in single-cell and single-molecule experiments respectively) and the coloured area (red and grey for SpsD and ΔSpsD cells respectively) from the standard deviation from this mean value.

Supplementary Table 1. Mean adhesion force value and calibration value for single-cell experiments for each cell.

Cell	Mean adhesion force (pN)	Cantilever spring constant (N/m)	
**1**	1900	0.12	Cantilever1
**2**	1899	0.12	
**3**	2012	0.12	
**4**	1954	0.12	
**5**	1908	0.12	
**6**	1727	0.095	Cantilever2
**7**	1686	0.095	
**8**	1624	0.095	
**9**	1747	0.095	Cantilever3
**10**	1863	0.095	
**11**	1836	0.099	Cantilever4
**12**	1862	0.099	
**13**	1717	0.099	
**14**	1719	0.099	
**15**	1725	0.099	

Supplementary Table 2. Mean adhesion force value and calibration value for single-molecule experiments for each cell.

Cell	Mean adhesion force (pN)	Cantilever spring constant (N/m)	
**1**	1537	0.022	Cantilever1
**2**	1458	0.022	
**3**	1484	0.022	
**4**	1391	0.022	Cantilever2
**5**	1604	0.022	
**6**	1589	0.022	
**7**	1604	0.032	Cantilever3
**8**	1704	0.017	Cantilever4
**9**	1551	0.051	Cantilever5
**10**	1542	0.051	
**11**	1653	0.051	

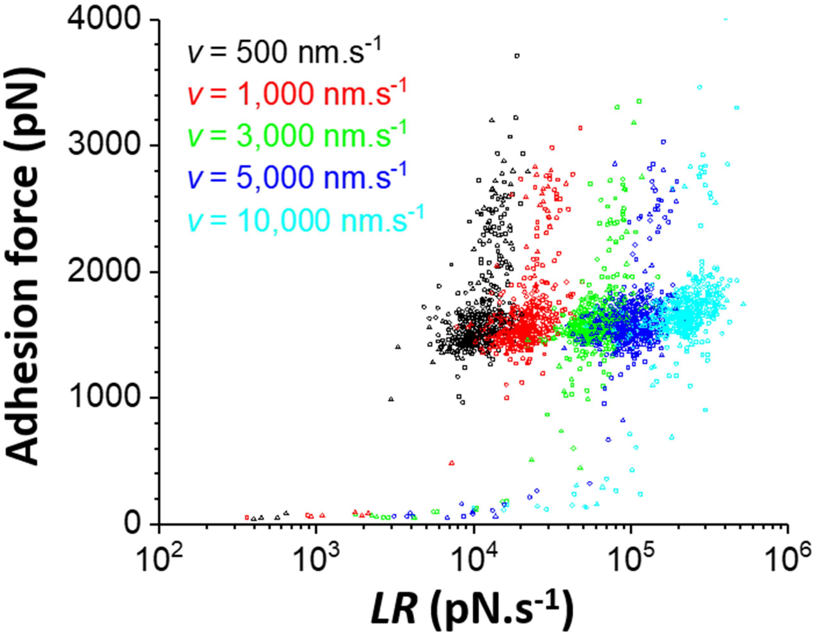

Supplementary Figure 5. Dynamic force spectrum on three merged SpsD cells and color-coded depending on the retraction velocities *v*.

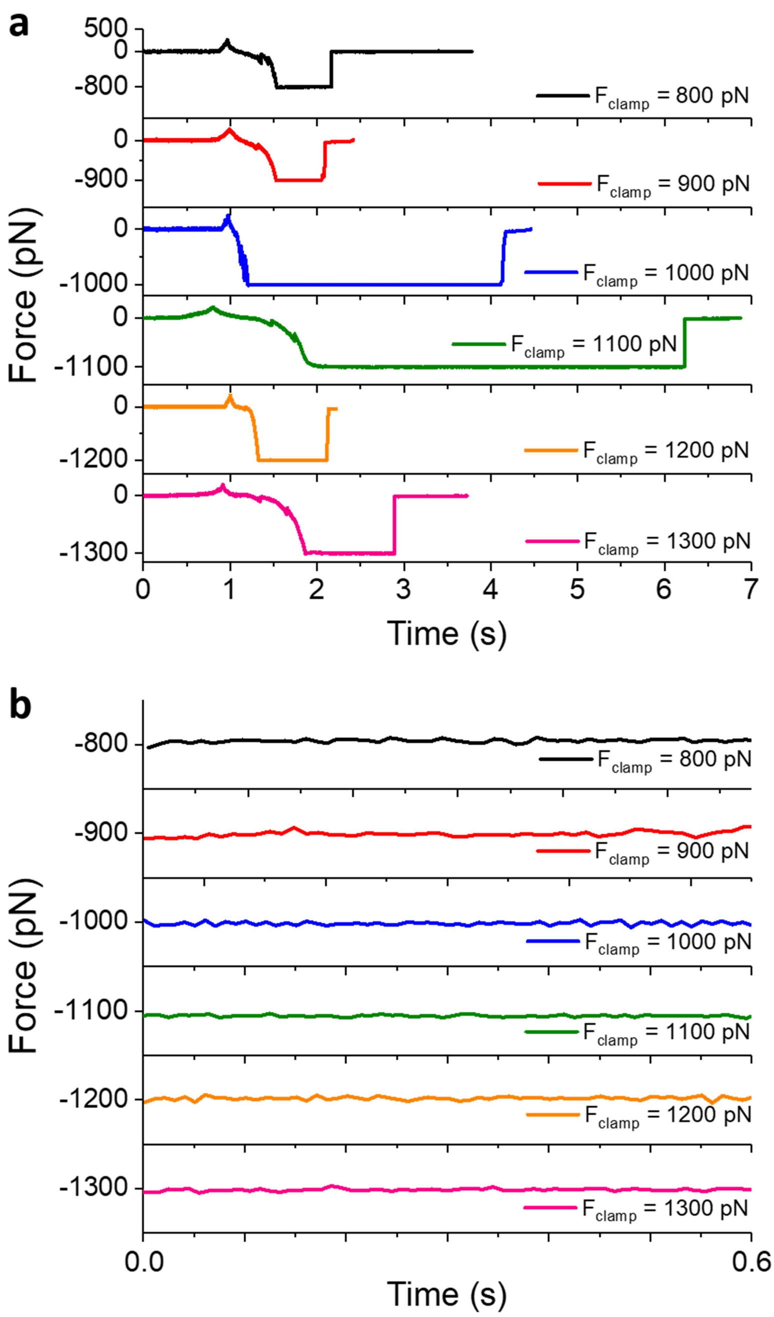

Supplementary Figure 6. (a) Representative force vs time curves obtained at difference clamping forces, and at a retraction velocity of 1,000 nm.s^{-1}, suggesting a force-dependence for the SpsD-fibrinogen lifetime. (b) Zoom in on the force vs time curves in the clamping region showing no significant deviation from the central value. The force scale and time scales are kept constant for each clamping force, 100 pN and 0.6 s respectively.

Supplementary Table 3. Fitting parameters obtained by fitting the survival plots at different clamping force by a single exponential decay.

	Lifetime (s) (mean ± standard error)			X^2	R^2
800 pN	0,514	±	0,003	$1,2.10^{-4}$	0,998
900 pN	0,645	±	0,015	$1,1.\ 10^{-3}$	0,969
1,000 pN	1,797	±	0,031	$1,1.\ 10^{-3}$	0,975
1,100 pN	2,330	±	0,017	$1,3.10^{-4}$	0,994
1,200 pN	1,179	±	0,016	$5,7.10^{-4}$	0,986
1,300 pN	0,797	±	0,013	$8,1.10^{-4}$	0,983

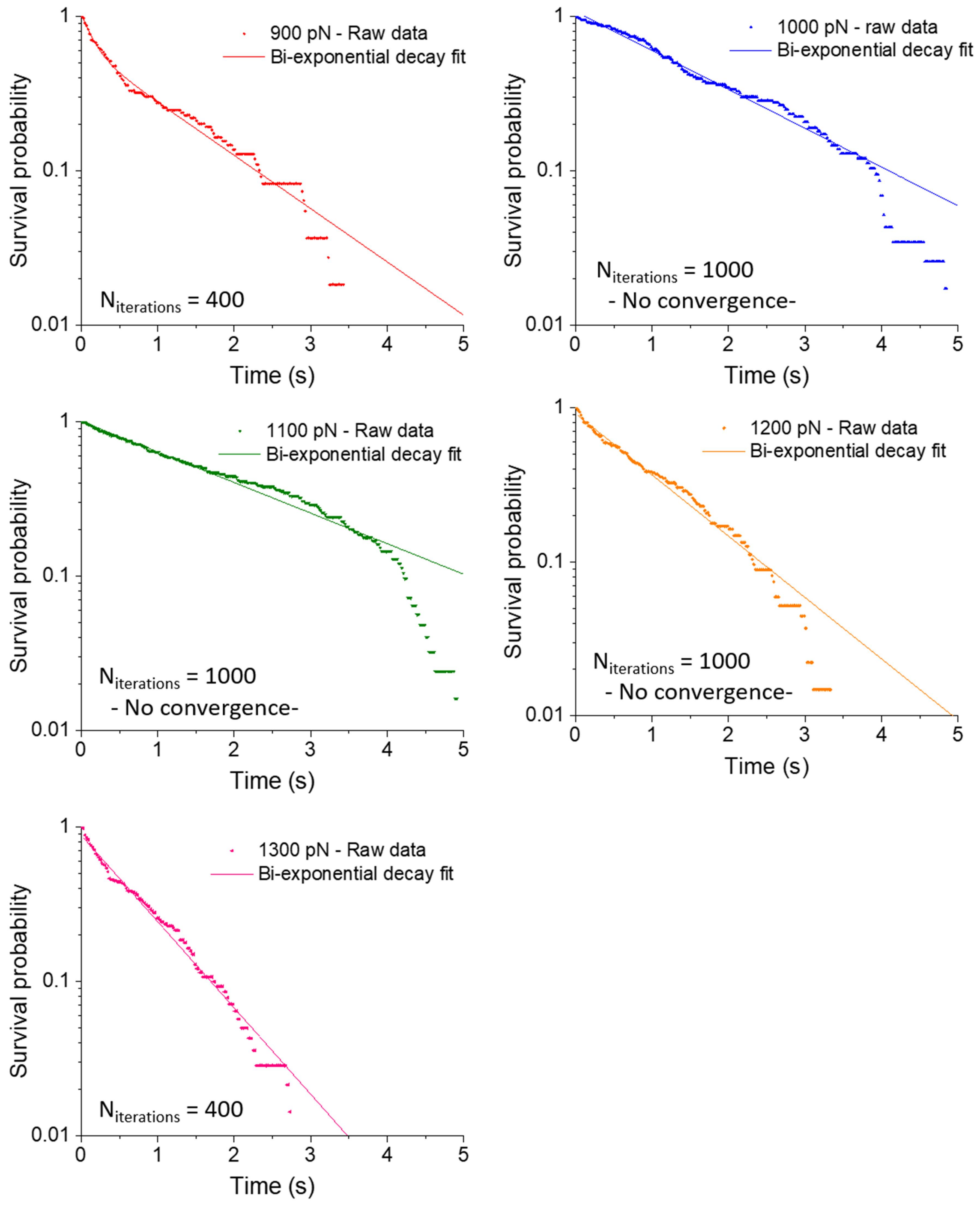

Supplementary Figure 7. The high strength involved in the SpsD-fibrinogen catch bond cannot be explained by classical bi-exponential decays. Bond survival probabilities for the SpsD-fibrinogen interactions, measured at 5 different clamping forces. Bi-exponential fits are presented as dashed lines with corresponding color. The number of iterations used and the fit convergence is also mentioned.

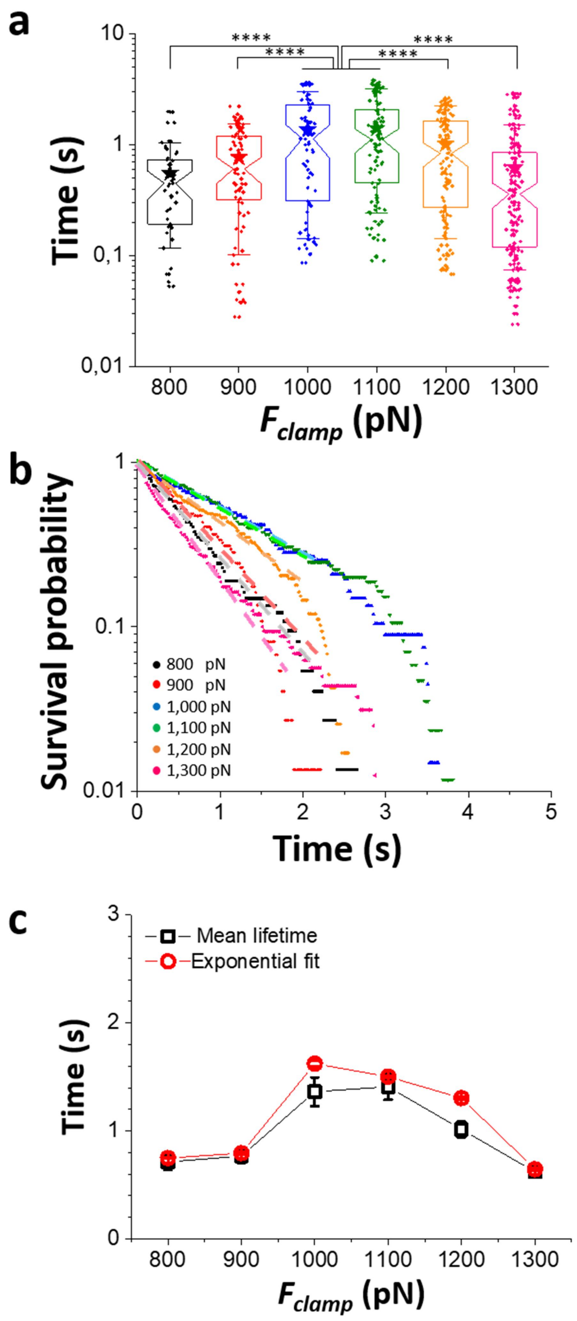

Supplementary Figure 8. Ramp rate does not influence the SpsD-fibrinogen bond lifetime. (a) Box plot (overlapped with data, n = 74, 74, 67, 85, 117 and 160 for F_{clamp} = 800, 900, 1,000, 1,100, 1,200, 1,300 pN respectively recorded on 11 independent cells from 6 independent cultures) of the lifetimes extracted from the force vs time curves at a retraction velocity of 10,000 nm.s^{-1}. Stars are the mean values, boxes the 25-75 % quartiles and whiskers the 10-90 % interval. Kruskal–Wallis test followed by Dunn's multiple-comparison test: **** $p \leq 0.0001$. (b) Bond survival probabilities for the SpsD-fibrinogen interaction obtained by clamping at difference loads and at a retraction velocity of 10,000 nm.s^{-1}. Fits are presented as dashed lines with corresponding colours. (c) Force-dependent bond lifetimes, extracted from the exponential fit on the survival plot and the average lifetimes from the box plot, at a retraction velocity of 10,000 nm.s^{-1}, exhibit a catch-slip transition. For

average lifetimes, error bars represent the standard deviation from n = 74, 74, 67, 85, 117 and 160 for F_{clamp} = 800, 900, 1,000, 1,100, 1,200, 1,300 pN respectively, on 11 independent cells from 6 independent cultures (as in (a)). For lifetimes extracted from the exponential fit, error bars stand for the standard error of the fit.
